# Macrophage membrane-coated polydopamine nanomedicine for treating acute lung injury through modulation of neutrophil extracellular traps and M2 macrophage polarization

**DOI:** 10.1016/j.mtbio.2025.101708

**Published:** 2025-03-24

**Authors:** Yuwei Zhao, Xingyu Zhu, Letao Hu, Fangyu Hao, Xianglei Ji, Xiaofang Hu, Meimei Luo, Linyu Zheng, Bo Xiao, Yingmei Wu, Changcan Shi, Hui Zhu, Nong Zhou, Weidong Li

**Affiliations:** a School of Pharmacy, Nanjing University of Chinese Medicine, Nanjing, 210023, China; b Chongqing College of Traditional Chinese Medicine, Chongqing, 402760, China; c Chongqing Three Gorges University, Chongqing, 402020, China

**Keywords:** Macrophage membrane-coated nanomedicine, Drug delivery system, Acute lung injury, Neutrophil extracellular traps, M2 polarization

## Abstract

Acute lung injury (ALI) is a life-threatening pulmonary inflammatory disorder with high morbidity and mortality rates. Effective targeting of damaged lung tissues and regulation of inflammatory dysregulation are major challenges in clinical treatment. This study aimed to develop a multifunctional drug delivery system by coating mesoporous polydopamine nanoparticles (mPDA NPs) loaded with Peimine (PM) using macrophage membranes (MMs) to leverage their inflammatory targeting properties. Both in vitro and in vivo experiments demonstrated the excellent targeting capability, strong antioxidant activity, and significant anti-inflammatory effects of the developed MM@mPDA-PM NPs. Furthermore, transcriptomics analysis revealed that MM@mPDA-PM NPs significantly reduced myeloperoxidase (MPO), neutrophil elastase (NE), and peptidylarginine deiminase 4 (PAD4), as well as inhibited the formation of neutrophil extracellular traps (NETs), and promoted M2 macrophage polarization by downregulating the NF-κB and JAK/STAT pathways. Our developed system effectively reduced neutrophil infiltration, suppressed cytokine storms, and regulated the pulmonary immune microenvironment, demonstrating great potential for treating ALI and other inflammatory diseases.

## Highlights

• The macrophage membrane-coated mPDA nanoparticles loaded with Peimine effectively target acute lung injury for treatment.
• MM@mPDA-PM nanoparticles demonstrate strong antioxidant and anti-inflammatory effects both in vitro and in vivo.
• The nanoparticles inhibit neutrophil extracellular trap formation by lowering MPO, NE, and PAD4 expression.
• MM@mPDA-PM nanoparticles promote M2 macrophage polarization and immune regulation by modulating NF-κB and JAK/STAT pathways.

## Graphical abstract

**Figure:**
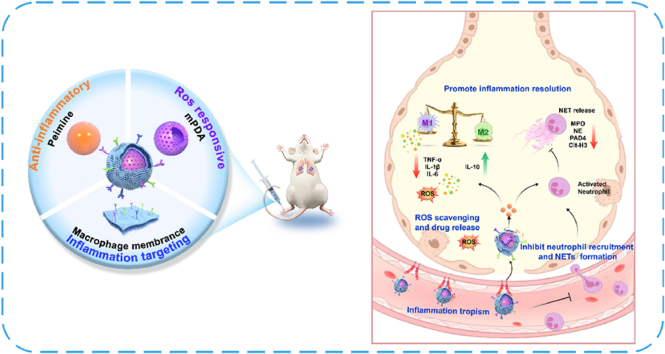

## 1 Introduction

Acute lung injury (ALI) and its more advanced counterpart, acute respiratory distress syndrome (ARDS), are marked by the breakdown of the alveolar-capillary membrane barrier and a runaway inflammatory response [1,2]. These conditions strike suddenly, wreaking havoc on the lungs' ability to function properly. Roughly 2 % of COVID-19 patients develop ALI or acute respiratory distress syndrome (ARDS), which can drive the mortality rate for these conditions as high as 90 % [3]. The effectiveness of current treatments for ALI/ARDS is a topic of ongoing debate [4]. Take glucocorticoids, for example—while they're sometimes used, their broad immunosuppressive effects and side effects can do more harm than good for certain ARDS patients, without offering any long-term benefits [5]. This underscores the urgent need for targeted, effective therapies to better manage ALI/ARDS.

In the context of ALI, alveolar macrophages play a crucial role in driving inflammatory reactions by releasing various cytokines and chemokines [6]. This process triggers the persistent influx of numerous neutrophils and monocytes, ultimately causing a disruption in the regulation of inflammation. In addition to releasing toxic reactive oxygen species (ROS) into tissues, neutrophils also release pro-inflammatory, pro-angiogenic, and pro-fibrotic mediators, exacerbating organ damage and prolonging inflammation [7]. When pro-inflammatory cytokines and damage-associated molecular patterns activate neutrophils, they prompt the release of neutrophil extracellular traps (NETs) [8]. These NETs are intricate, web-like structures made up of unraveled chromatin and proteins from neutrophil granules [9]. The creation of these traps doesn't just stop there—it amplifies lung damage and fuels inflammatory reactions, worsening conditions like ALI and acute respiratory distress syndrome (ARDS) [10]. In essence, NETs play a pivotal role in driving the vicious cycle of injury and inflammation in these critical conditions [11]. Moreover, ineffective drug delivery to lung tissues reduces therapeutic efficacy in ALI/ARDS [12]. Effective targeting of the lung injury site, ROS and neutrophilic trap inhibition, and macrophage M2 polarization regulation could improve the immune microenvironment, serving as a potential therapeutic strategy for ALI/ARDS treatment.

Biologically derived cell membrane-coated NPs have attracted considerable attention owing to their in vivo cell-like simulation potential and natural targeting capabilities [13]. Both neutrophils and macrophages exhibit significant inflammatory tropism. However, large-scale production and widespread therapeutic application of neutrophils is limited by their short lifespan (a normal life span in blood circulation is approximately 8–10 h) [14]. In addition to their critical function in the immune system, macrophages are key players in the inflammatory process [15]. They possess a natural attraction to chemokines, facilitated by receptors like C-C chemokine receptor type 2 (CCR2), C-C motif chemokine receptor 6 (CCR6), and C-X-C Motif Chemokine Receptor 1 (CXCR1), which are embedded in macrophage membranes [16]. These receptors act as molecular gateways, prompting macrophages to latch onto areas of inflammation or tumor growth, where they contribute to the body's defense mechanisms [17]. A recent study by Simon and colleagues demonstrated that NPs encapsulated in macrophage membranes (MM) could outperform macrophages containing internalized NPs in addressing inflammatory diseases [18]. This research underscores the considerable promise of cell membrane-coated NPs as a cutting-edge therapeutic approach. Zhang et al. described a multiantigen nano-toxin vaccine utilizing MM-encapsulated NPs to address inflammatory lung infections [19]. The innate inflammation-directed chemotactic ability of macrophages promotes vector accumulation in inflammatory tissue, exhibiting superior targeting ability.

Peimine (PM)—one of the most active components of Bulbus Fritillariae cirrhosae—is an important anti-inflammatory herb in traditional Chinese medicine [19]. PM is widely used as a therapeutic agent for cough and asthma owing to its high efficacy and minimal side effects [20]. The literature indicates its anti-inflammatory properties. For instance, PM suppressed IL-1β-induced inflammation in mouse chondrocytes by repressing the MAPK pathway, thereby alleviating osteoarthritis [21]. Additionally, it downregulated inflammatory cytokines and upregulated anti-inflammatory cytokines in LPS-stimulated RAW 264.7 macrophages [22]. Liu et al. [23] further demonstrated the synergistic anti-inflammatory effect of a combination of PM, peiminine, and forsythoside in the treatment of LPS-induced ALI through the IL-17-NF-κB/MAPK pathway inhibition. Nevertheless, the clinical use of PM is limited because of its water insolubility, short half-life, and low bioavailability [24]. Therefore, the development of a reliable carrier system to enhance the delivery of hydrophobic PM is crucial for its broader therapeutic potential.

Nanomedicine has been investigated as a method for controlled drug delivery, including natural compounds, to reduce side effects in ALI/ARDS treatment [25]. Mesoporous polydopamine (mPDA) NPs are particularly promising due to their rich pore structures and surface functionalized sites, which enable effective drug adsorption, storage, and controlled release [26]. mPDA NPs exhibit efficient loading capacity for hydrophobic drugs, enhancing their bioavailability. In addition, mPDA NPs possess excellent ROS scavenging capabilities, which can help mitigate the oxidative stress associated with ALI/ARDS [27].

**Scheme 1:**
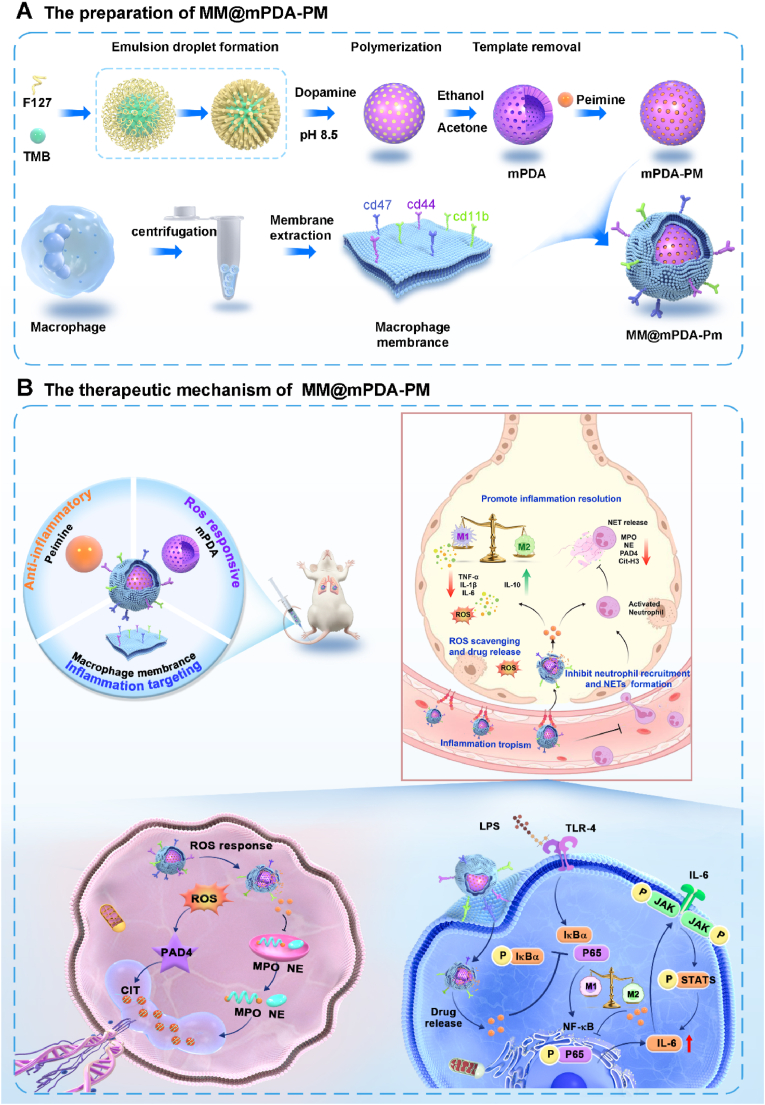
Schematic illustration for the preparation of MM@mPDA-PM NPs and the therapeutic mechanism against Acute lung injury. (A) the preparation of MM@mPDA-PM NPs. (B) the therapeutic mechanism of MM@mPDA-PM NPs.

In this study, a multifunctional drug delivery system, termed MM@mPDA-PM NPs was constructed by coating mesoporous polydopamine (mPDA) loaded with Peimine (PM) with macrophage membranes (MM), leveraging the inflammatory tropism of macrophages (Scheme 1). This system exhibited excellent inflammation-targeting capabilities. In an ALI model, MM@mPDA-PM NPs achieved remarkable therapeutic effects. MM@mPDA-PM NPs significantly reduced myeloperoxidase (MPO), neutrophil elastase (NE), and peptidylarginine deiminase 4 (PAD4) levels, inhibited the formation of NETs, and demonstrated robust antioxidant and anti-inflammatory effects. Simultaneously, MM@mPDA-PM NPs downregulated the NF-κB and JAK/STAT pathways, promoted M2 macrophage polarization, modulated the immune microenvironment of lung tissue, reduced neutrophil infiltration, and suppressed cytokine storms. This study offers a promising drug delivery platform for the targeted treatment of inflammation-related diseases.

## 2 Materials and methods

### 2.1 Materials

Peimine was purchased from Shizhou Biology (Nanjing, China). Dopamine hydrochloride, Pluronic F-127, Acetone, and LPS were purchased from Aladdin Reagents (Shanghai, China). 1,3,5-trimethylbenzene (TMB), Membrane and Cytosol Protein Extraction kit, BCA assay kit, CCK-8, ROS assay kit, DAPI, One-Step TUNEL Cell Apoptosis Detection kit, 5-Fluorescein isothiocyanate (FITC), DID were purchased from Beyotime (Shanghai, China). MPO assay kit, SOD assay kit, and MDA assay kit were purchased from Nanjing Jiancheng Bioengineering Institute. ELISA kits were purchased from Dakewe Biotech Co., Ltd. (China). Antibodies used for immunofluorescence, Western blot, and FCM detection were sourced from Abcam, Cell Signaling Technology. All other chemicals were used without further puriffcation.

### 2.2 Synthesis of the mPDA-PM NPs

To synthesize mPDA NPs, we started by combining 1 g of F127 with 0.5 g of DA hydrochloride in a 100 mL solution of ethanol and deionized water, maintaining a 1:1 ratio. Gradually, we introduced 2 mL of TMB, and the mixture was stirred magnetically for 30 min until a white emulsion developed. Once that process was complete, we added 5 mL of NH_3_·H_2_O and stirred the mixture vigorously for 24 h. Afterward, we centrifuged the solution at 13,000 rpm for 10 min and proceeded to wash the residue three times using an ethanol and acetone mixture in a 2:1 ratio, applying ultrasonic agitation for 30 min during each wash. Finally, the product was suspended in water for subsequent applications.

To load PM into mPDA, 2 mg of PM was first dissolved in 1 mL of ethanol. This solution was then combined with 1 mL of an mPDA dispersion (1 mg/mL, ethanol-soluble) under ultrasonic conditions. The resulting mixture was stirred continuously at room temperature for 6 h, followed by centrifugation and thorough washing to yield the final product, mPDA-PM NPs.

### 2.3 Synthesis of MM@mPDA-PM NPs

MMs were extracted from RAW264.7 cells using a well-established protocol, albeit with a slight adjustment. The cell membrane of RAW264.7 cells was isolated by employing a membrane protein extraction kit, adhering strictly to the guidelines provided by the manufacturer. To conclude, the collected cells were immersed in a buffer solution designed for membrane protein extraction and placed on ice for about 15 min. Subsequently, the cell mixture was gently transferred to a glass homogenizer, where it underwent approximately 30 cycles of homogenization. Following this, the resulting homogenate underwent centrifugation—first at 1500 rpm for 10 min at 4 °C, and then at 14000 rpm for an additional 30 min to separate out the cell membranes. To determine the total protein content in the isolated PMs, a bicinchoninic acid (BCA) protein assay was carried out. To prepare MM vesicles, the isolated MMs underwent a 15 min sonication process, followed by extrusion through a 400-nm polycarbonate membrane using an Avestin mini extruder, repeated 10 times. The resulting MM vesicles were then collected and kept in water at 4 °C. To coat the NPs with the MMs, the extracted vesicles were mixed with the mPDA-PM NPs (1:1 wt ratio). The mixture was forced through a 200-nm polycarbonate membrane, and the resulting NPs coated with cell membranes were kept at 4 °C for later use.

### 2.4 Colocalization study

The MMs were stained with DiD, while the mPDA NPs were stained with FITC. The DiD-labeled MMs were directly extruded onto the mPDA NPs, following the previously detailed method. To investigate colocalization after cellular uptake, HUVECs were grown in DMEM enriched with 10 % fetal bovine serum and maintained at 37 °C in a 5 % CO_2_ environment. MM@mPDA was introduced to the HUVECs, which were then allowed to incubate for 4 h at 37 °C. Afterward, the cells were thoroughly rinsed three times with phosphate-buffered saline (PBS) to remove any residual material. They were subsequently fixed using a tissue fixative for 30 min at room temperature. To wrap things up, the nuclei were stained with DAPI for another 30 min, maintaining the same temperature and conditions throughout the process. Subsequent to this, a confocal laser scanning microscopy (CLSM) technique was employed to visualize the cells.

### 2.5 Cellular uptake

The capacity of cells to escape was initially evaluated in standard macrophages. A suitable quantity of these cells was plated in 12-well dishes and allowed to culture overnight. The existing medium was then swapped for a new solution containing mPDA and MM@mPDA. After incubation for 1, 2, 3 and 4 h, the uptake levels of mPDA and MM@mPDA in the cells were measured using fluorescence-activated cell sorting (FACS) analysis. To visualize the cell nuclei, DAPI staining was employed, and the uptake process was monitored through CLSM. In an effort to replicate an inflammatory cell model, macrophages and HUVECs were stimulated with LPS at a concentration of 100 ng/mL. Following this activation, the same procedures were carried out to assess the targeting efficiency of MM@mPDA within the inflammatory cells.

### 2.6 Intracellular ROS assessment

Raw 264.7 cells were plated into 24 - well plates at a density of 2 × 10^5^ cells per well and cultured overnight under inflammatory stimulation conditions. Subsequently, the existing culture medium was replaced with a fresh medium containing different pharmaceuticals. The cells were divided into the following experimental groups: (1) Control; (2) H_2_O_2_; (3) H_2_O_2_ + Free PM; (4) H_2_O_2_ + mPDA; (5) H_2_O_2_ + mPDA-PM; (6) H_2_O_2_ + MM@mPDA-PM. After a 4-h incubation period, 0.5 mL of DCFH-DA was added for an additional 30 min of incubation, after which the cells were rinsed twice. Subsequently, ROS levels were assessed using fluorescence microscopy and flow cytometry (FCM).

### 2.7 In vitro anti-inflammatory analysis

To evaluate the anti-inflammatory effects of the NPs, RAW264.7 cells were seeded into 24-well plates at a density of 2 × 10^5^ cells per well and cultured overnight under LPS (1 μg/mL) stimulation. Subsequently, the medium was replaced with formulations containing MM@mPDA-PM NPs or controls. After 12 h, supernatants were harvested, and cytokine levels (TNF-α, IL-1β, IL-6, and IL-10) were measured using ELISA kits (R&D Systems, USA).

### 2.8 Macrophage polarization effect

RAW264.7 cells were initially plated in a 6-well plate in accordance with of the density of 1 × 10^6^ cells/well and then grouped and managed accordingly. After 24 h, the cells were collected and centrifuged, with the supernatant being removed. The cells underwent three thorough washes with PBS to ensure cleanliness, and their concentration was fine-tuned to 1 × 10^6^ cells per milliliter. Next, the cells were incubated with anti-mouse CD86-FITC (for M1 macrophages) and anti-mouse CD206-PE (for M2 macrophages) at 4 °C for 30 min in the dark. Subsequently, the cells were washed, centrifuged, and suspended in a new solution. Flow cytometry was then used to detect and analyze the cells. M1 and M2 macrophage expressions were determined based on the proportion of CD86^+^ and CD206+ cells.

### 2.9 Animal models

Male BALB/c mice were acquired from Qing Long Shan Animal Breeding Farm and maintained in a specific-pathogen-free facility. All experimental procedures were conducted in accordance with the protocols approved by the Nanjing University of Chinese Medicine Animal Care and Use Committee (202403A069).

### 2.10 In vivo distribution

The BALB/c male mice model was initially developed by administering endotracheal aerosolized LPS at a concentration of 5 mg/mL in PBS. To evaluate their in vivo distribution, mPDA and MM@mPDA were tagged with a near-infrared fluorescent dye (Cy7.5 at a dosage of 0.5 mg/kg) and subsequently injected intravenously 4 h post-LPS exposure. At specified time points, the primary organs were harvested and analyzed using an in vivo imaging system (IVIS).

### 2.11 In vivo anti-inflammatory effectiveness: therapeutic administration

BALB/c mice were anesthetized by intraperitoneal injection of 1 % sodium pentobarbital. To establish the lung injury model, endotracheal aerosolized LPS (5 mg/mL in HBSS) was delivered. 4 h after the induction of acute lung injury (ALI), the mice were allocated into different treatment groups: PBS, free PM, mPDA-PM, and MM@mPDA-PM. At specific time intervals, following two administrations at the scheduled times, experiments were carried out on the lung tissue to evaluate the anti-inflammatory effectiveness in vivo.

### 2.12 Analysis of lung tissue homogenate

Upon treatment completion, lung tissues were flash-frozen in liquid nitrogen and maintained at −80 °C for subsequent analysis. Lung tissues were homogenized to detect superoxide dismutase (SOD) activity, as well as malondialdehyde (MDA) and MPO levels. The homogenate was centrifuged at 12000 g for 10 min, and the resulting supernatant was assessed with the appropriate kits.

### 2.13 Histological analysis

Lung tissue alterations were evaluated through hematoxylin and eosin (H&E) staining. Immunohistochemistry (IHC) staining for Ly6G was performed to assess neutrophil infiltration. Immunofluorescence (IF) staining of dihydroethidium (DHE) was performed to evaluate ROS production in tissues. Additionally, IF staining for CD206 and iNOS was conducted to evaluate pro- and anti-inflammatory substance secretion in tissues.

### 2.14 Statistical analysis

Statistical analysis was performed using a two-tailed Student's *t*-test and one-way analysis of variance (ANOVA). Data are presented as the mean ± standard deviation (SD). Statistical significance is indicated as ∗P < 0.05, ∗∗P < 0.01, ∗∗∗P < 0.001, and ∗∗∗∗P < 0.0001.

## 3 Results and discussion

### 3.1 MM@mPDA-PM NP synthesis and characterization

**Fig. 1:**
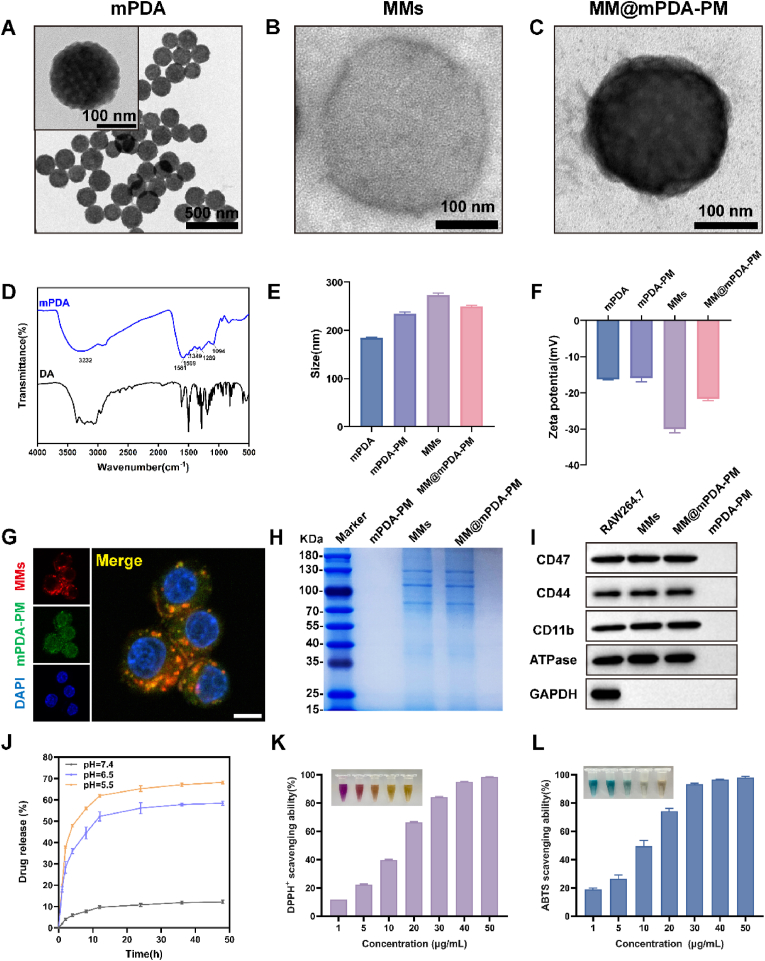
**Synthesis and characterization of MM@mPDA-PM NPs.** TEM images of (A) mPDA NPs, (B) MMs (RAW264.7 cell membrane), and (C) MM@mPDA-PM NPs. (D) Fourier transform infrared spectra of PDA. (E, F) Hydrodynamic diameters and zeta potential of mPDA, mPDA-PM, MMs, and MM@mPDA-PM NPs (n = 3). (G) CLSM images of the cellular uptake of fluorescence-labeled MM@mPDA-PM NPs (red, MMs; green, mPDA-PM). (H) SDS–PAGE protein analysis of mPDA-PM, MMs, and MM@mPDA-PM NPs (I) Representative western blot analysis of CD47, CD44, CD11b, adenosine triphosphatase (ATPase), and glyceraldehyde-3-phosphate dehydrogenase (GAPDH) in RAW264.7, MMs, MM@mPDA-PM NPs, and mPDA-PM groups. (J) Peimine release curves of MM@mPDA-PM NPs incubated in simulated medium (PBS with different pH). (K) DPPH and (L) ABTS scavenging ratio of MM@mPDA-PM NPs. Data are expressed as the mean ± SD.

mPDA was prepared using the soft template method (Scheme 1). Subsequently, PM was loaded onto the mPDA to produce mPDA-PM NPs. Finally, MMs were coated onto the surface of mPDA-PM NPs to form membrane-coated mPDA-PM NPs—MM@mPDA-PM NPs—through repeated extruding processes. Transmission electron microscopy (TEM) images showed abundant surface mesopores and voids in the mPDA, offering ample space for drug encapsulation (Fig. 1A). Fourier transform infrared (FTIR) spectroscopy analysis further validated the successful synthesis of mPDA (Fig. 1D). FTIR spectra of dopamine (DA) showed typical small molecule features, whereas the mPDA NPs exhibited several characteristic absorption peaks at approximately 1500–1600 and 1100-1350 cm^−1^. These peaks demonstrated the decrease in aromatic hydrogen and nuclei in mPDA NPs, confirming the polymer structure's successful formation. Due to the abundance of functional groups, mPDA can effectively load drugs through π-π interactions. PM was added into an ethanol solution of mPDA and stirred for 6 h to achieve successful drug loading. The excess drug was then removed via ultrafiltration. The PM loading content, determined using the liquid chromatography–mass spectrometry (LC-MS), was approximately 34.4 %, with encapsulation efficiency of approximately 68.2 %. To enhance the stability and precision of nanoparticle targeting, MMs were isolated from the RAW264.7 mouse macrophage cell line using a probe-based ultrasound technique. Following this, MM vesicles were carefully prepared through a series of steps involving ultracentrifugation, thorough washing, and ultrasonication. Transmission electron microscopy (TEM) analysis of the purified MM vesicles confirmed the presence of a well-defined lipid bilayer structure (Fig. 1B). Next, PM-loaded mPDA (mPDA-PM) was coextruded with MM vesicles to construct the MM-coated mPDA-PM NPs (MM@mPDA-PM NPs). The surface of the NPs, coated with MMs, showed distinct light-colored ring-shaped structures, indicating the successful coating of MMs (Fig. 1C).

To validate the successful coating of MMs on the surface of mPDA-PM NPs, the MMs and mPDA-PM NPs were labeled with DiD (red) and FITC (green) fluorescent dyes and imaged using CLSM. Yellow coloration observed on overlapping the two images indicated the presence of green and red signals within the same particle, confirming successful coating of MMs on mPDA-PM NPs (Fig. 1G). Membrane proteins were analyzed using sodium dodecyl sulfate–polyacrylamide (SDS-PAGE) gel electrophoresis assay (Fig. 1H). The results revealed a significant presence of RAW264.7 cell membrane proteins in the MM@mPDA-PM NPs, indicating the effective incorporation of cell membranes onto the NPs. Interestingly, the mPDA-PM NPs showed no detectable protein signal, which strongly suggests that the membrane proteins remained intact following the physical extrusion process. Additionally, western blot analysis confirmed the presence of critical cell surface markers, including CD47—a key regulator of macrophage phagocytosis—as well as CD44 and CD11b, which play essential roles in targeting mechanisms (Fig. 1I). CD11b and CD44 possess the capacity to interact with intercellular adhesion molecule-1 and P-selectin, molecules that are highly expressed on the surface of activated endothelial cells [28,29]. Consequently, chemokine receptors and adhesion molecules collaborate seamlessly with membrane proteins to guide the precise movement of NPs toward inflammatory sites. According to dynamic light scattering analysis, the hydrodynamic diameter of MM@mPDA-PM NPs increased by approximately 15 nm compared to their uncoated counterparts, while the zeta potential remained consistent with that of the original MM vesicles. This strongly suggests the successful encapsulation of mPDA within the MM@mPDA-PM structure (Fig. 1 E, F). Next, the stability of the NPs was investigated in simulated physiological conditions. The hydrodynamic diameter and zeta potential of MM@mPDA-PM in PBS and 10 % fetal bovine serum demonstrated the stability of the prepared nanomedicine under physiological conditions (Fig. S1).

### 3.2 ROS scavenging and PM release of MM@mPDA-PM NPs

ALI and similar inflammatory conditions advance, ROS are produced to help modulate the inflammatory response [30]. However, when ROS levels spiral out of control, they can wreak havoc on lung tissue, leading to significant damage. By effectively neutralizing this excess ROS, it's possible to mitigate the severity of ALI and curb inflammation in the lungs. We assessed the catalytic efficiency of MM@mPDA-PM NPs with a range of substrates, such as 1-diphenyl-2-picrylhydrazyl (DPPH) and ABTS radicals. Remarkably, when 50 μg/mL of MM@mPDA-PM NPs was introduced, over 95 % of both DPPH and ABTS were reduced, highlighting their exceptional capability for ROS scavenging. (Fig. 1K and L; Fig. S2).

During ALI progression, the local microenvironment of lung injury tissue becomes weakly acidic, with excessive ROS production [31]. Building on these findings, we delved deeper into the antioxidant capabilities of mPDA NPs and the pH-responsive drug release behavior of PM. Given that mPDA is synthesized through polymerization under alkaline conditions, it stands to reason that the carrier would break down and release its payload in the slightly acidic environment typical of inflammatory conditions. This dual functionality makes mPDA NPs a promising candidate for targeted therapeutic applications. In PBS media at pH of 7.4, 6.5, and 5.5, the cumulative drug release of MM@mPDA-PM NPs at 24 h was 10.8 %, 56.1 %, and 65.2 % for PM, respectively (Fig. 1J). This suggests that in mildly acidic environments, the breakdown of nanocarriers accelerates the swift delivery of drugs directly into the diseased tissue. At a neutral pH of 7.4, the drug release from MM@mPDA-PM NPs remained below 13 % over a 48 h period, highlighting the system's ability to curb premature drug leakage during systemic circulation. This controlled release mechanism minimizes toxicity and spares healthy tissues from unintended harm, showcasing the precision of the nanomedicine.

### 3.3 Characterization of the immune-evasive functions

For a drug delivery system to achieve its efficacy, it is crucial that it be absorbed by the intended target cells [32]. This is the key to ensuring optimal drug performance while minimizing collateral damage to other tissues and organs [33]. To achieve this, one of the key benchmarks is making sure the system can dodge the macrophages of the reticuloendothelial system, which are notorious for gobbling up foreign particles. In other words, steering clear of phagocytosis is essential when evaluating the system's effectiveness. Emerging evidence indicates that MM-coated NPs reduce macrophage-mediated phagocytosis.

**Fig. 2:**
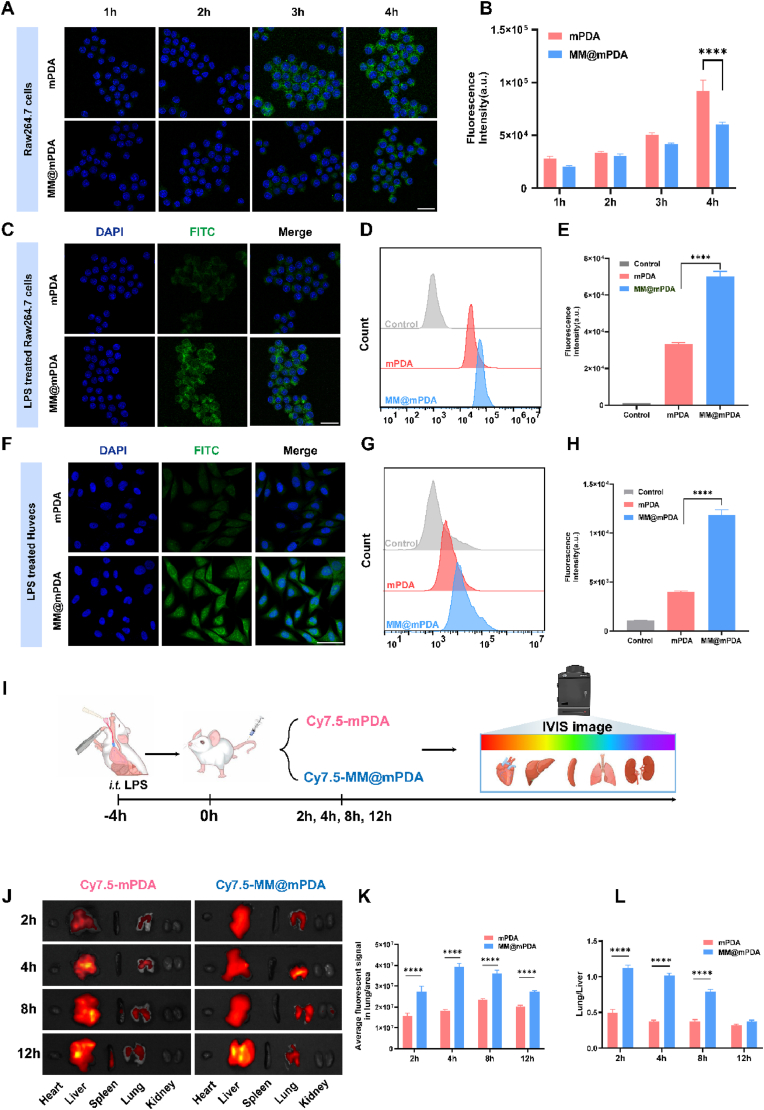
**Inflammation targeting properties of MM@mPDA NPs.** (A, B) Ability to escape from mouse mononuclear macrophage leukemia (RAW264.7) cells (including flow cytometry quantitative analysis) of free mPDA and MM@mPDA NPs. Scale bar: 25 μm. (C) Fluorescence images of cellular uptake of mPDA and MM@mPDA NPs by RAW264.7 cells treated with lipopolysaccharide (LPS). Scale bar: 25 μm. (D, E) FACS results and quantification of cellular uptake of mPDA and MM@mPDA NPs in RAW264.7 cells. (F) Fluorescence images showing cellular uptake of mPDA and MM@mPDA NPs by human umbilical vein endothelial cells (HUVECs) treated with LPS. Scale bar: 25 μm. (G, H) FACS results and quantification of cellular uptake of mPDA and MM@mPDA NPs in HUVECs. (I) Schedule for the experiments in (J)−(L). (J) Ex vivo biodistribution of Cy7.5-mPDA, and Cy7.5-MM@mPDA in main organs at different time intervals via IVIS imaging. (K) Fluorescent semiquantitation in the lungs (n = 3). (L) Fluorescent semiquantitation of the lung-to-liver ratio of each group (n = 3). Data are expressed as the mean ± SD. Data were assessed using one-way ANOVA and two-way ANOVA; ∗∗∗∗P < 0.0001.

We assessed the uptake of MM@mPDA NPs in RAW264.7 cells. CLSM images indicated that macrophages internalized both mPDA NPs and MM@mPDA NPs over time. However, the green fluorescence intensity from mPDA NPs was stronger than that from MM@mPDA NPs at the same time points (Fig. 2A), indicating greater mPDA NPs internalization. This was further confirmed via FACS analysis, where the internalization content of mPDA NPs was approximately 1.38, 1.11, 1.21, and 1.52 times higher than that of the MM@mPDA NPs after 1, 2, 3, and 4 h of incubation, respectively (Fig. 2B; Fig. S3). These results suggest that the MM coating on NPs effectively reduces nonspecific phagocytosis by macrophages.

### 3.4 Targeted delivery in vitro

Our study focused on evaluating the targeted delivery capabilities of MM@mPDA NPs toward inflammatory cells under stress conditions. To mimic an inflammatory environment, RAW264.7 cells were stimulated with LPS. We then assessed the cellular uptake of both mPDA NPs and MM@mPDA NPs using CLSM for visualization and FACS for quantitative analysis. The CLSM results revealed a marked increase in the uptake of MM@mPDA NPs by activated macrophages, as evidenced by more intense green fluorescence signals, compared to their mPDA NPs counterparts (Fig. 2C). FACS analysis further confirmed this, demonstrating 2.1-fold higher uptake of MM@mPDA NPs in activated macrophages than that of mPDA NPs (Fig. 2D and E). This phenomenon may be attributed to LPS-induced upregulation of macrophage membrane receptors (Fcγ receptors and scavenger receptors) and enhanced phagolysosomal activity, collectively improving the recognition and internalization of MM@mPDA NPs. To further verify the targeting ability of MM@mPDA NPs, MM@mPDA NPs uptake was observed in activated HUVECs (Fig. 2F, G, H), which play a major role in inflammation initiation. CLSM images and quantitative FACS analysis revealed that MM decoration on MM@mPDA NPs enhanced the cellular uptake of MM@mPDA NPs in activated endothelial cells. Collectively, these results demonstrate that the MM@mPDA NPs effectively target inflammatory cells and facilitate drug delivery to the inflammation site. Furthermore, The chemotactic properties of the MM proteins guide the NPs to areas of inflammation, enabling precise drug release. The findings indicate that the MM coating allows MM@mPDA NPs to cleverly dodge macrophage uptake, thereby improving their ability to zero in on inflammatory cells. This implies that MM@mPDA NPs can deliver drugs with pinpoint accuracy directly to inflamed areas, steering clear of unintended side effects elsewhere.

### 3.5 In vivo targeting

Given the remarkable in vitro capability of MM@mPDA NPs to home in on inflammatory cells, we decided to take things a step further and explore their potential to specifically target inflamed lungs in live mice. To evaluate the lung-targeting prowess of mPDA NPs and MM@mPDA NPs, we employed near-infrared fluorescence IVIS imaging at various intervals within the ALI mouse model. This approach allowed us to track their behavior and effectiveness in real-time, shedding light on their therapeutic potential. (Fig. 2I). Cy7.5-MM@mPDA-labeled NPs showed significantly greater accumulation in the lungs compared to their non-membrane-coated counterparts (Cy7.5-mPDA NPs), 2 h post administration (Fig. 2J and K). This enhanced targeting is attributed to the rapid clearance of the liver, where mPDA NPs mainly accumulate following whole-body injection. 2 h after injection, the fluorescence signal of the lung-to-liver ratio was elevated from 0.50 (mPDA) to 1.12 (MM@mPDA) (Fig. 2L). Overall, these findings indicate that MM@mPDA NPs efficiently target and accumulate in lung inflammatory sites.

### 3.6 In vitro antioxidant activity of MM@mPDA-PM NPs

**Fig. 3:**
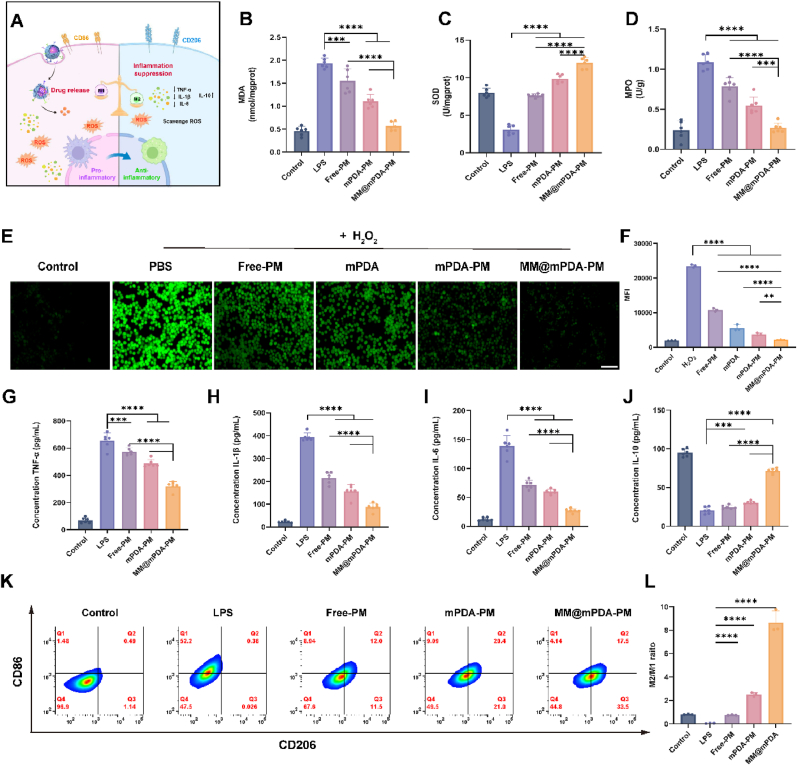
**In Vitro Anti-inflammatory Effects of MM@mPDA-PM NPs.** (A) Illustration for the experiments in (B)−(L). The levels of (B) MDA, (C) SOD and (D) MPO in the supernatant of inflamed macrophages after treatment (n = 6). (E) Fluorescence images and (F) flow cytometry analysis of ROS after treatments in RAW 264.7 cells (n = 3), scale bar: 50 μm. Cytokine levels of (G) TNF-α, (H) IL-1β, (I) IL-6 and (J) IL-10 in the supernatant of inflamed macrophages after treatment (n = 6). (K) Investigation of the phenotypic polarization of macrophages. FCM was used to detect the polarization of inflamed RAW267.4 after treatment with Free-PM, mPDA-PM, and MM@mPDA-PM NPs, respectively. RAW267.4 stimulated without LPS served as the control group (n = 3). (L) Quantitative representation of the M2/M1 ratio after treatments. Data are expressed as the mean ± SD. Data were assessed using one-way ANOVA and two-way ANOVA; ∗∗P < 0.01, ∗∗∗P < 0.001, ∗∗∗∗P < 0.0001.

To delve deeper into the ROS clearance within cells, we triggered RAW264.7 cells with H_2_O_2_ to prompt the generation of intracellular ROS. The intracellular ROS levels across various treatment groups were quantitatively assessed using fluorescence microscopy and flow cytometry following a 4 h exposure period. The cells with higher ROS generation (detected using 2ʹ-7ʹdichlorofluorescin diacetate) should exhibit higher fluorescence. The capacity of various treatments to scavenge ROS was evaluated through the measurement of fluorescence signals from the ROS probe. In comparison to the PBS group, the Free-PM group showed a minor reduction in fluorescence intensity. On the other hand, the mPDA, mPDA-PM, and MM@mPDA-PM NP groups demonstrated a significant decrease in ROS levels, which highlights the enhanced intracellular ROS scavenging efficacy of mPDA (Fig. 3E and F; Fig. S4). Furthermore, MM@mPDA-PM NPs exhibited the best ROS-scavenging effect. Subsequently, we assessed SOD activity and MDA and MPO levels in RAW264.7 cells following LPS treatment. Compared with the LPS group, SOD activity was significantly upregulated (Fig. 3C) and MDA (Fig. 3B) and MPO (Fig. 3D) levels were downregulated in other treatment groups (all P < 0.001). Among treatment groups, the results for MM@mPDA-PM NPs closely resembled those of the control group, demonstrating their antioxidant capacity. Collectively, these findings demonstrate the effective antioxidative capability of MM@mPDA-PM NPs.

### 3.7 In vitro anti-inflammatory effects of MM@mPDA-PM NPs

Oxidative stress significantly influences macrophage polarization in diseases associated with inflammation. To assess the effects of MM@mPDA-PM NPs, we performed an in vitro study. Inflammatory macrophages were used to evaluate the in vitro anti-inflammatory effect of MM@mPDA-PM NPs, with unstimulated macrophages as a control. ELISA revealed that the pro-inflammatory factors (TNF-α and IL-6) were significantly reduced, whereas the inflammatory suppressor (IL-10) was elevated in the treatment groups (all P < 0.001; Fig. 3G–J). Macrophages exhibit remarkable plasticity, adapting their functional roles depending on the microenvironment they inhabit. These adaptations are grouped into two phenotypes: classically activated (M1) and alternatively activated (M2) macrophages. M1 macrophages are primarily associated with driving inflammatory processes, while M2 macrophages are pivotal in modulating immune responses and facilitating tissue repair. To induce the M1 phenotype, we stimulated RAW264.7 cells with LPS, a well-established activator for inducing M1 macrophage polarization. After the induction of LPS, there was an observable increase in the M1 phenotype (CD86^+^), and it became clear that the predominant population of inflammatory macrophages was M1. Following this, M1 macrophages were treated with Free-PM, mPDA-PM, and MM@mPDA-PM NPs for a duration of 24 h. The subsequent assessment of their anti-inflammatory properties involved analyzing the M1 inflammatory phenotype and quantifying pro-inflammatory cytokine levels, both of which are crucial markers for determining the shift of macrophages from a pro-inflammatory to an anti-inflammatory phenotype. Flow cytometry analysis of CD86, a characteristic biomarker of M1 macrophages, was used to assess this transition. The gated analysis from flow cytometry of treated macrophages is shown in Fig. 3K and Fig. S5. Compared to the Free-PM group, the M1 subpopulation (CD86^+^) decreased, whereas the M2 subpopulation (CD206+) increased in the MM@mPDA-PM NPs group. The M2-to-M1 ratio in the MM@mPDA-PM NPs group increased from 0.73 (Free PM) to 8.65 (MM@mPDA-PM NPs) (Fig. 3L). The results suggest that MM@mPDA-PM NPs exhibit anti-inflammatory effects, potentially due to enhanced cellular uptake by inflammatory macrophages, which enhances the effect of PM. Taken together, these results suggest that MM@mPDA-PM NPs have he ability to reprogram inflammatory macrophages, steering them toward an anti-inflammatory, reparative phenotype under laboratory conditions. This shift not only reduces the production of proinflammatory mediators but also increases the presence of anti-inflammatory macrophages, underscoring the promising therapeutic applications of MM@mPDA-PM NPs.

### 3.8 MM@mPDA-PM NPs exhibit an antioxidative effect on LPS-induced ALI mice

**Fig. 4:**
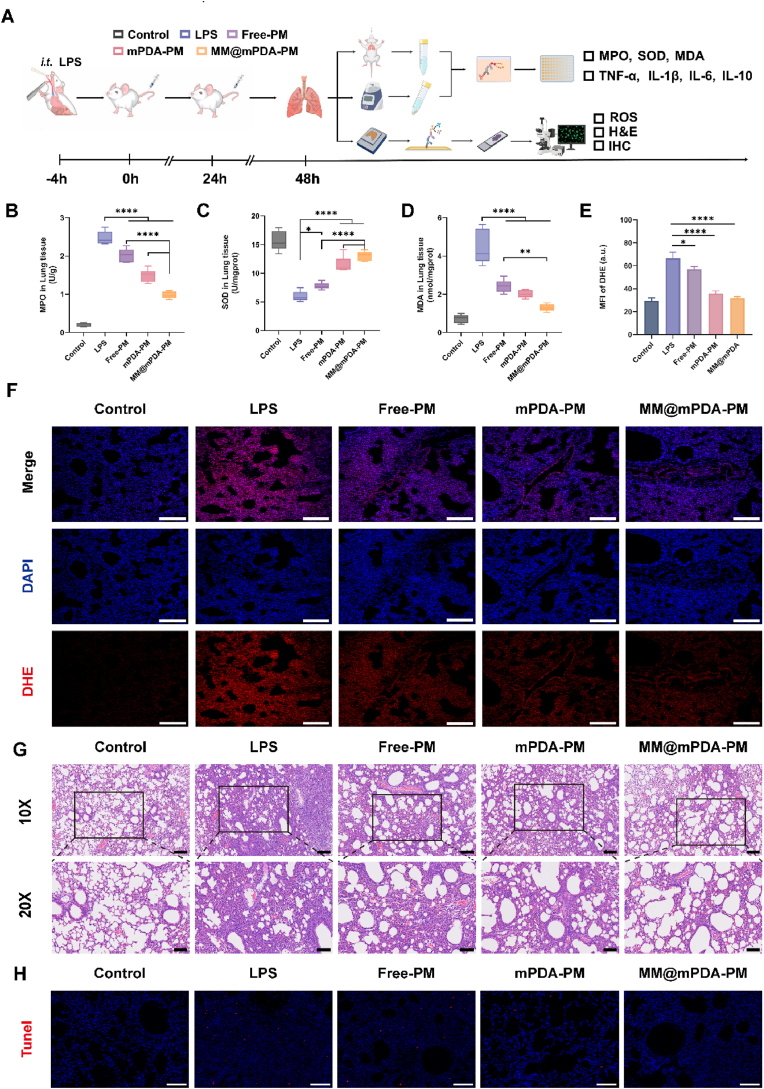
**MM@mPDA-PM NPs restore LPS-induced lung damage and attenuate the elevated ROS level in ALI mice.**(A) Schematic of the experimental design for the ALI model study. The ALI model was established by intratracheal administration of LPS (5 mg/kg). At 4 h after the LPS challenge, mice were intravenously treated with normal saline, Free-PM, mPDA-PM, and MM@mPDA-PM NPs. The lung tissues or BALF were obtained for therapeutic analysis at 48 h post administration. (B) MPO, (C) SOD and (D) MDA levels of lung tissue homogenate (n = 6). (E, F) Quantification results and representative fluorescence images visualizing DHE secretion (n = 3). (G) H&E staining images of lung sections. (H) TUNEL staining in lung tissue. Scale bars: 100 μm. Data are expressed as the mean ± SD. Data were assessed using one-way ANOVA and two-way ANOVA; ∗P < 0.05, ∗∗P < 0.01, ∗∗∗∗P < 0.0001.

Having verified the ROS - scavenging potential of MM@mPDA - PM NPs in vitro, we then evaluated their antioxidant properties in vivo. This was done using a model of ALI triggered by LPS. To induce ALI, we administered LPS (5 mg/kg) directly into the lungs via endotracheal atomization. At 4 h post-modeling, normal saline, Free-PM, mPDA-PM, and MM@mPDA-PM NPs were intravenously injected into the mice. For distinct experimental and pathological assessments, bronchoalveolar lavage fluid (BALF) and lung tissues were retrieved 24 h subsequent to the second dosage administration. (Fig. 4A). MPO, SOD, and MDA are key markers of oxidative stress and antioxidant defense [34]. MPO, a peroxidase predominantly generated by neutrophils, is linked to tissue damage during acute inflammatory responses due to the presence of excessive MPO-derived oxidants [35]. MDA, the main byproduct of lipid oxidation, serves as a key marker for oxidative stress. Meanwhile, SOD is crucial for modulating ROS levels and offers protective in mice with LPS-induced ALI [36]. MM@mPDA-PM NPs treatment significantly decreased MPO and MDA levels in lung tissues, with the most significant downregulation in the MM@mPDA-PM NPs group (Fig. 4B–D). Furthermore, compared to the LPS group, SOD was significantly upregulated in the MM@mPDA-PM NPs group (Fig. 4C). We then performed DHE staining of lung tissues to visualize ROS production. Compared to the saline-treated mice, the generation of ROS in the MM@mPDA-PM NPs group was markedly inhibited. This suggests that MM@mPDA-PM NPs might be able to scavenge ROS, ease oxidative stress, and inhibit the development of ALI (Fig. 4E and F).

Next, the protective effects of MM@mPDA-PM NPs on lung injury and host cell damage were further assessed using H&E staining (Fig. 4G) and terminal deoxynucleotidyl transferase-mediated deoxyuridine triphosphate–biotin nick end labeling (TUNEL) (Fig. 4H). Severe lung injury, characterized by extensive inflammatory cell infiltration, pulmonary edema, and alveolar wall thickening, was observed in LPS-induced ALI mice. However, treatment with MM@mPDA-PM NPs alleviated lung injury in the ALI mice. The TUNEL assay consistently demonstrated heightened levels of apoptosis following LPS-induced damage. However, the MM@mPDA-PM NPs treatment group showed a marked decrease in TUNEL-positive cells, as evidenced by the reduction in red signals. This suggests that MM@mPDA-PM NPs effectively mitigate apoptosis under these conditions. These results collectively demonstrate that MM@mPDA-PM NPs reduce oxidative stress and cellular apoptosis, effectively alleviating LPS-induced ALI in mice.

### 3.9 Anti-inflammatory efficacy of MM@mPDA-PM NPs in vivo

**Fig. 5:**
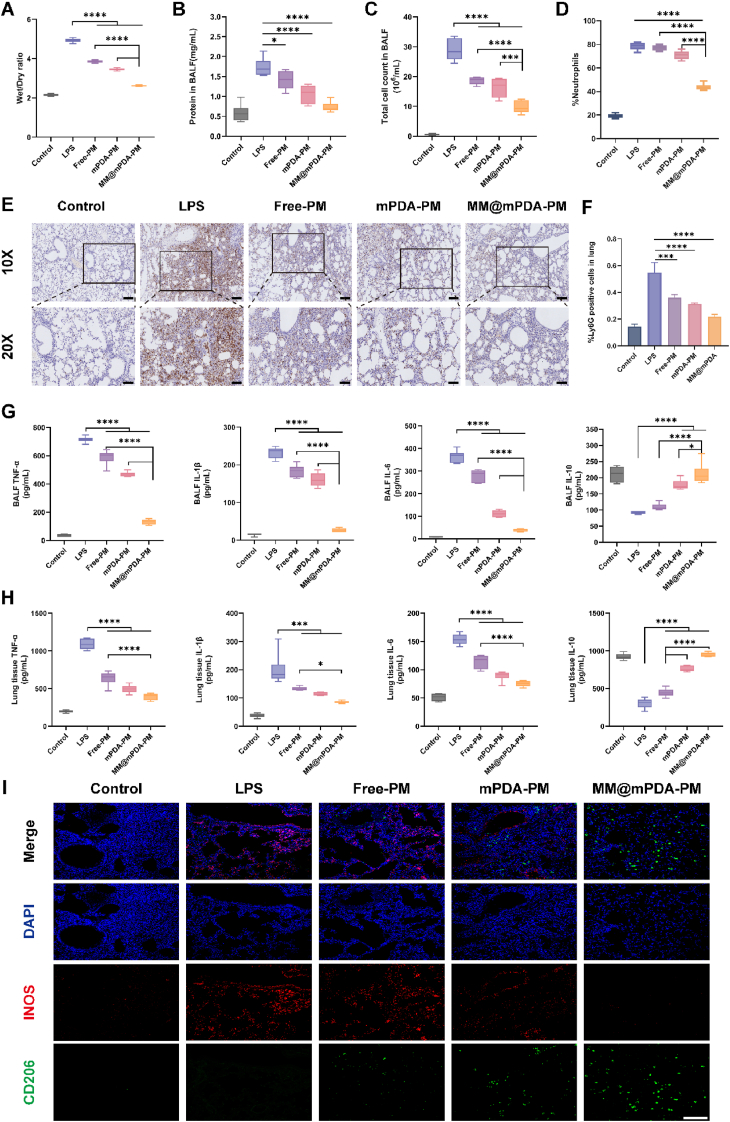
**In vivo anti-inflammatory efficiency of MM@mPDA-PM NPs.**(A) The W/D ratio of lungs following various treatments (n = 6). (B) Protein content, (C) total cell count, and (D) quantitative analysis of neutrophil proportion in BALF lung tissue after various treatments (n = 6). (E) IHC analysis for Ly6G expression, scale bars: 100 μm. (F) The quantitative results of Ly6G positive cells in IHC images (n = 3). (G) TNF-α, IL-1β, IL-6 and IL-10 levels in BALF of different groups (n = 6). (H) TNF-α, IL-1β, IL-6 and IL-10 levels in lung tissues of different groups (n = 6). (I) IF images of iNOS and CD206 expression in lungs from ALI model mice. Scale bar: 200 μm. Data are expressed as the mean ± SD. Data were assessed using one-way ANOVA and two-way ANOVA; ∗P < 0.05, ∗∗P < 0.01, ∗∗∗∗P < 0.0001.

The underlying mechanism of ALI revolves around widespread inflammation, which triggers cellular damage, heightened capillary permeability, and the buildup of fluid in the interstitial spaces, alongside the migration of inflammatory cells into the affected area [37]. To evaluate how effective MM@mPDA-PM NPs are in treating this condition, we analyzed pulmonary edema by calculating the wet-to-dry (W/D) weight ratio and measuring protein levels within the lungo tissue. The LPS group exhibited a higher W/D ratio than the control group (Fig. 5A). However, the W/D ratio decreased in the treatment groups, with the most significant decrease in the MM@mPDA-PM NPs group. Additionally, the protein concentration in the lungs tissues was reduced in the MM@mPDA-PM NPs group compared to the LPS group (Fig. 5B). The findings indicate that MM@mPDA-PM NPs have the potential to mitigate edema and the leakage of interstitial fluid in the ALI model. Neutrophils and macrophages are crucial players in the advancement of ALI, with neutrophil levels in the lungs serving as a key indicator of the inflammation's intensity [38]. Successful ALI therapies focus on curbing the excessive recruitment of neutrophils, modulating proinflammatory macrophage activity, and restoring a balanced inflammatory environment [39]. Consequently, our initial step involved analyzing the infiltrating cells within the lungs (Fig. 5C). The total cell count in the BALF of mice increased significantly following LPS challenge, which was approximately 54-fold higher than that of healthy mice, and decreased after treatment with Free-PM, mPDA-PM, and MM@mPDA-PM NPs. Furthermore, we observed a marked reduction in neutrophil recruitment in mice treated with MM@mPDA-PM NPs compared to the LPS group (Fig. 5D). The enhanced ability of MM@mPDA-PM NPs to specifically target the lungs may account for their more pronounced impact on inhibiting leukocyte recruitment compared to mPDA-PM. Additionally, these NPs demonstrated a significant decrease in both total cell and neutrophil counts when compared to Free-PM, suggesting that MM-targeted delivery leads to greater PM effectiveness. Supporting this conclusion, immunohistochemical staining for the Ly6G neutrophil marker revealed a notable reduction in neutrophil infiltration within the lung tissues of mice treated with MM@mPDA-PM NPs (Fig. 5E and F).

Cytokine storms exacerbate the inflammatory cascade in the lungs and are closely associated with mortality in patients with ALI [40]. To investigate whether MM@mPDA-PM NPs can attenuate lung inflammatory cytokine storms in mouse models of lung injury, we measured the levels of TNF-α, IL-1β, IL-6, and IL-10 in blood serum, BALF, and lung tissue using ELISA. Treatment with Free-PM, mPDA-PM, or MM@mPDA-PM NPs reduced TNF-α, IL-1β and IL-6 levels (Fig. 5G and H; Fig. S6). Nonetheless, the inhibitory impact was somewhat constrained. On the other hand, the group treated with MM@mPDA-PM NPs showed a notable decrease in the levels of pro-inflammatory cytokines, closely aligning with the levels observed in healthy mice. These results imply that MM@mPDA-PM NPs can significantly suppress the release of pro-inflammatory cytokines in a living organism. Additionally, treatment with MM@mPDA-PM NPs encouraged the production of the anti-inflammatory cytokine IL-10.

We also assessed the levels of the pro-inflammatory marker iNOS and the anti-inflammatory marker CD206 in lung cells through immunofluorescence (IF) staining. The results revealed that MM@mPDA-PM NPs effectively reduced iNOS secretion while boosting CD206 expression (Fig. 5I). This suggests that MM@mPDA-PM NPs play a role in modulating the inflammatory environment in the lungs, tipping the balance toward a more anti-inflammatory state. Overall, MM@mPDA-PM NPs demonstrated excellent potential in inhibiting neutrophil recruitment, attenuating lung inflammatory cytokine storms, and improving the overall inflammatory microenvironment in the ALI model.

### 3.10 Biosafety assessment of MM@mPDA-PM NPs

**Fig. 6:**
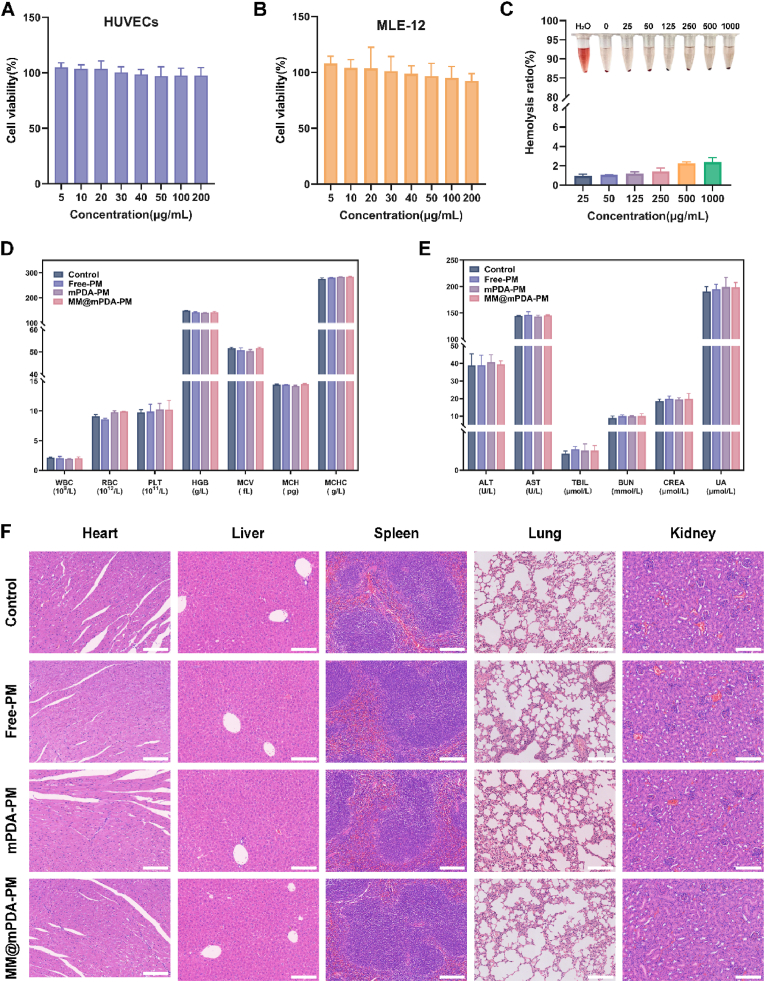
**Biocompatibility of MM@mPDA-PM NPs.** (A, B) Cell viability of HUVEC and MLE-12 cells incubated for 24 h with varying concentrations of MM@mPDA-PM NPs (n = 6, mean ± SD). (C) Photograph and corresponding rate of hemolysis assay of MM@mPDA-PM NPs in different concentrations. (D) Typical hematological parameters (n = 3, mean ± SD). (E) Biochemical markers relevant to hepatic and kidney function (n = 3, mean ± SD) (F) Representative H&E images for vital organs from mice at 1 week after intravenous injection of different reagents. Scale bar: 100 μm.

Since toxic side effects on normal organs and the entire system are concerns during drug treatment, we conducted both in vitro and in vivo assessments [41]. The in vitro cytotoxicity of MM@mPDA-PM NPs was first evaluated by assessing the viability of HUVECs and MLE-12 following MM@mPDA-PM NPs treatment (Fig. 6A and B). MM@mPDA-PM NPs did not significantly affect cell viability at the tested concentrations. Additionally, the assessment of the blood compatibility of MM@mPDA-PM NPs revealed excellent hemocompatibility with no significant hemolytic reaction in red blood cells (Fig. 6C).

For the in vivo biosafety assessment, healthy mice were randomly assigned to three groups (n = 3) and injected intravenously with saline, Free-PM, mPDA-PM, or MM@mPDA-PM NPs. After a week, key organs—including the heart, lungs, liver, spleen, and kidneys—along with blood samples were gathered for thorough examination. H&E staining and hematological tests revealed no significant changes in white blood cells (WBC), red blood cells (RBC), platelets (PLT), hemoglobin (HGB), mean corpusular volume (MCV), mean corpusular hemoglobin (MCH), and mean corpusular hemoglobin concentration (MCHC) (Fig. 6D). Clinical biochemistry analysis revealed that the levels of alanine aminotransferase (ALT), aspartate aminotransferase (AST), total bilirubin (TBIL), blood urea nitrogen (BUN), creatinine (CREA), and uric acid (UA) remained within normal ranges. This finding suggested that the biological functions of the liver and kidney were not influenced by the treatment. (Fig. 6E). Moreover, the H&E-stained sections of all organs in the treatment groups showed no major pathological alterations (Fig. 6F). These findings further confirm the excellent biocompatibility of MM@mPDA-PM NPs, supporting their potential for therapeutic applications.

### 3.11 Transcriptomic analysis and validation of the treatment effects of MM@mPDA-PM NPs on alleviating cytokine storm in pneumonia

**Fig. 7:**
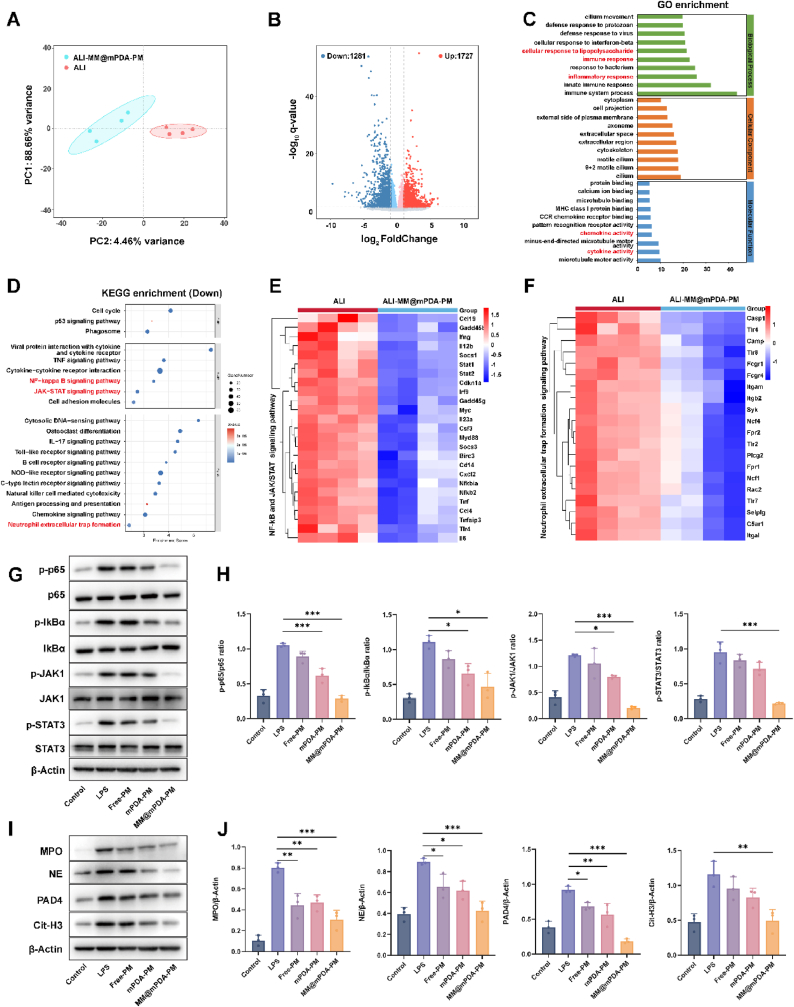
**Therapeutic mechanisms of MM@mPDA-PM NPs on calming cytokine storm**(A) Principal component analysis (PCA) of RNA-seq datasets across ALI-MM@mPDA-PM NPs and ALI groups. Each data point describes the PCA analysis of each sample. (B) Volcano plot showing differentially expressed genes in ALI-MM@mPDA-PM NPs group versus ALI group. (C) Bar plot of GO enrichment analysis of the top 10 DEGs in Biological Process, Cellular Component and Molecular Function. (D) KEGG pathways enrichment analysis of the top 20 downregulated DEGs by bubble diagram. (E)The key DEGs of the NF-κB and JAK/STAT signaling pathway among ALI-MM@mPDA-PM NPs and ALI groups were clustered in heatmap through RNA-seq analysis. (F) The key DEGs of the Neutrophil extracellular trap formation signaling pathway among ALI-MM@mPDA-PM NPs and ALI groups were clustered in heatmap through RNA-seq analysis. (G, H) Western blot analysis of protein expression of key members in the NF-κB and JAK/STAT signaling pathways, including p-NF-κB, p65, NF-κB p65, p-IκB-α, IκB-α, p-JAK1, JAK1, p-STAT3, and STAT3. (I, J) Western blot analysis of protein expression of key members in NET formation signaling pathways, including MPO, NE, PAD4, and Cit-H3. Data are expressed as the mean ± SD, with n = 4 animals per group in A−F and n = 3 animals per group in G–J. Data were assessed using one-way ANOVA and two-way ANOVA; ∗P < 0.05, ∗∗P < 0.01, ∗∗∗P < 0.001.

To delve deeper into how MM@mPDA-PM NPs mitigate cytokine storms, a transcriptomic analysis was conducted on lung tissue from the LPS-induced ALI model following treatment with MM@mPDA-PM NPs. This approach aimed to uncover the underlying molecular pathways influenced by the intervention. The ALI group served as the control. Principal component analysis revealed clear separation between the MM@mPDA-PM NPs-treated ALI group and the untreated ALI group, with significant transcriptome reprogramming in response to ALI-MM@mPDA-PM NPs treatment (Fig. 7A). Volcano plots further revealed 1727 upregulated and 1281 downregulated genes in MM@mPDA-PM NPs-treated ALI mice (Fig. 7B). To delve into the regulatory functions of these genes in inflammation-related pathways, Gene Ontology (GO) and Kyoto Encyclopedia of Genes and Genomes (KEGG) enrichment analyses were carried out. The GO analysis highlighted that the differentially expressed genes (DEGs) were predominantly linked to the cellular reaction to LPS, immune and inflammatory responses, as well as chemokine and cytokine activities (Fig. 7C). Most DEGs were enriched in categories related to inflammatory responses to bacterial infections. Macrophages and neutrophils serve as vital components of the innate immune system, acting as frontline defenders against microbial threats and orchestrating inflammatory processes. To shed light on how MM@mPDA-PM NPs influence the dynamic interplay between alveolar macrophages and neutrophils, we performed KEGG pathway analysis on the transcriptomic data. This approach allowed us to delve deeper into the mechanisms underlying immune modulation mediated by these NPs. MM@mPDA-PM NPs treatment downregulated NET formation signaling pathways and various signaling pathways associated with macrophages, including the TNF signaling pathway, IL-17 signaling pathway, NF-κB signaling pathway, and JAK/STAT pathway (Fig. 7D). The signaling pathways of interest are intricately linked to the inflammatory reactions of M1 macrophages. These cells secrete cytokines and chemokines that trigger inflammation, subsequently resulting in tissue damage. An RNA sequencing analysis grouped the differentially expressed genes (DEGs) associated with the NF-κB, JAK/STAT, and NET signaling pathways in both the ALI-MM@mPDA-PM NP and ALI groups (Fig. 7E and F). These results suggest that MM@mPDA-PM NPs treatment suppresses the activation of these pathways and inhibits NET formation.

To confirm these findings, the phosphorylation levels of proteins in the NF-κB and JAK/STAT signaling pathways were assessed using western blotting. The LPS group exhibited significant phosphorylation of NF-κB p65, IκB-α, JAK1, and STAT3 in mouse lung tissues (Fig. 7G). However, Free-PM, mPDA-PM, and MM@mPDA-PM NPs treatment markedly inhibited the phosphorylation of NF-κB p65, IκB-α, JAK1, and STAT3 in synovium (Fig. 7H). Since LPS triggers NET formation, we also evaluated key neutrophil proteins associated with ALI. MPO, NE, PAD4, and citrullinated histone H3 (Cit-H3) are primary neutrophil proteins that serve as major markers of NETs. As anticipated, MPO, NE, PAD4, and Cit-H3 levels were upregulated in the lung tissue of ALI mice, whereas their levels were significantly lower in the MM@mPDA-PM NPs group (Fig. 7I and J). These results suggest that MM@mPDA-PM NPs alleviate inflammation by regulating inflammatory macrophages through the inhibition of NF-κB and JAK/STAT signaling pathways and preventing the formation of NETs.

## 4 Conclusion

In this study, we developed a multifunctional drug delivery system (MM@mPDA-PM NPs) by coating NPs using MMs to leverage their natural affinity for inflammation sites. This system demonstrated excellent inflammation-targeting capabilities. In an ALI model, MM@mPDA-PM NPs significantly reduced the levels of MPO, NE, and PAD4, inhibited the formation of NETs, and exhibited strong antioxidant and anti-inflammatory effects. Simultaneously, MM@mPDA-PM NPs downregulated the NF-κB and JAK/STAT pathways, promoted M2 macrophage polarization, modulated the immune microenvironment of lung tissue, reduced neutrophil infiltration, and suppressed cytokine storms. These findings highlight MM@mPDA-PM NPs as a promising drug delivery platform for targeted treatment of inflammation-related diseases such as stroke and rheumatoid arthritis.

## CRediT authorship contribution statement

**Yuwei Zhao:** Writing – review & editing, Writing – original draft, Validation, Investigation, Formal analysis, Data curation, Conceptualization. **Xingyu Zhu:** Validation, Investigation. **Letao Hu:** Investigation. **Fangyu Hao:** Investigation. **Xianglei Ji:** Validation. **Xiaofang Hu:** Validation. **Meimei Luo:** Validation. **Linyu Zheng:** Validation. **Bo Xiao:** Software. **Yingmei Wu:** Validation. **Changcan Shi:** Writing – review & editing, Conceptualization. **Hui Zhu:** Writing – review & editing. **Nong Zhou:** Funding acquisition. **Weidong Li:** Writing – review & editing, Conceptualization.

## Availability of data and materials

All data needed to evaluate the conclusions in the paper are present in the paper and/or the Supplementary Materials.

## Declaration of competing interest

The authors declare that they have no known competing financial interests or personal relationships that could have appeared to influence the work reported in this paper.

## Data Availability

Data will be made available on request.
