# Supplementary material for: Macrophage membrane-coated polydopamine nanomedicine for treating acute lung injury through modulation of neutrophil extracellular traps and M2 macrophage polarization
Source: Mater Today Bio. 2025 Mar 24;32:101708. doi: 10.1016/j.mtbio.2025.101708 (PMC11987672; doi:10.1016/j.mtbio.2025.101708)
Supplement: Multimedia component 1 [file mmc1.docx]

**Supporting Information**

**Macrophage membrane-coated polydopamine nanomedicine for treating acute lung injury through modulation of neutrophil extracellular traps and M2 macrophage polarization**

Yuwei Zhao^a^, Xingyu Zhu^a^, Letao Hu^a^, Fangyu Hao^a^, Xianglei Ji^a^, Xiaofang Hu^a^, Meimei Luo^a^, Linyu Zheng^a^, Bo Xiao^b^, Yingmei Wu^c^, Changcan Shi^a,^*, Hui Zhu^a,^*,Nong Zhou^c,^*, Weidong Li^a,^*

^a^ School of Pharmacy, Nanjing University of Chinese Medicine, Nanjing 210023, China

^b^ Chongqing College of Traditional Chinese Medicine, Chongqing 402760, China

^c^ Chongqing Three Gorges University, Chongqing 402020, China.

* Correspondence author.

Correspondence: [cshi@njucm.edu.cn](mailto:cshi@njucm.edu.cn) (C. Shi), [zhui_0826@njucm.edu.cn](mailto:zhui_0826@njucm.edu.cn) (H. Zhu), erhaizn@126.com(N. Zhou), liweidong0801@njucm.edu.cn (W. Li).


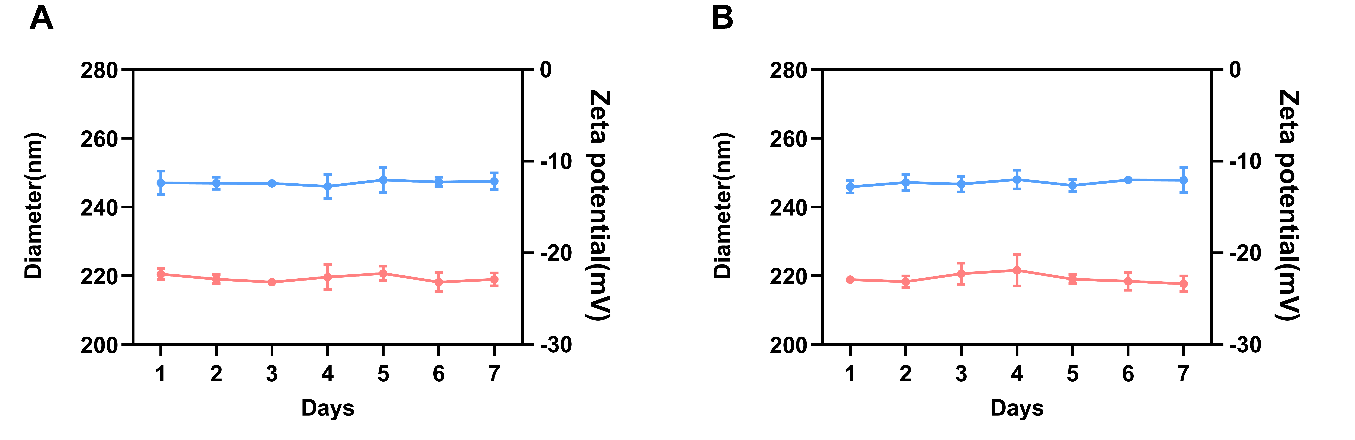


**Figure S1.** Particle size and zeta potential of MM@mPDA-PM NPs in PBS buffer (A) and 10% FBS (B) at different time. (n = 3). Data are expressed as the mean ± SD.


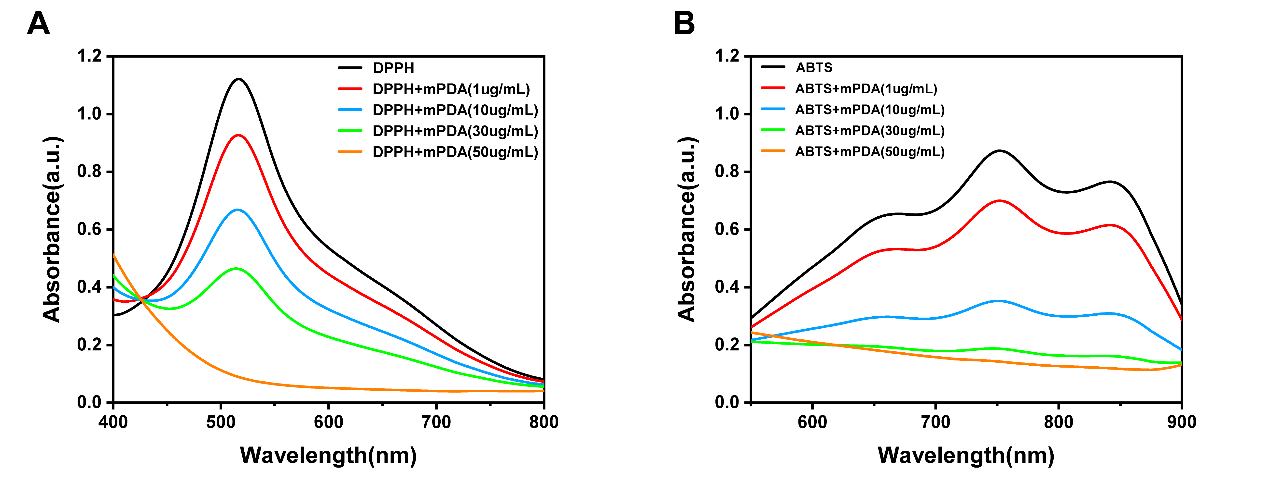


**Figure S2.** (A)UV–vis absorbance spectra of DPPH· radicals after incubation with the MM@mPDA-PM NPs.

(B) UV–vis absorbance spectra of ABTS+· radicals after incubation with MM@mPDA-PM NPs.


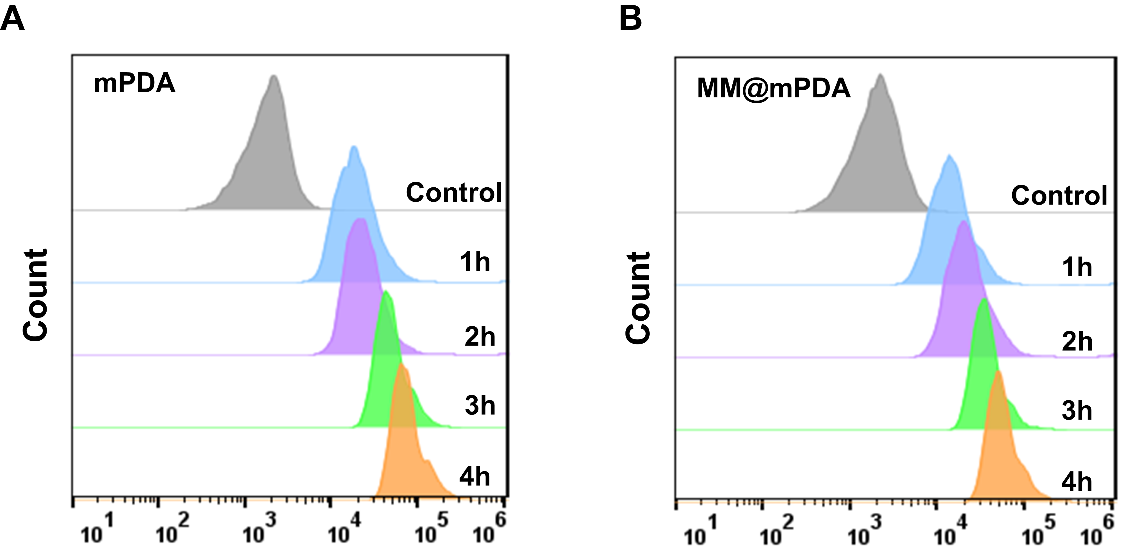


**Figure S3.** FACS results (processing for 1, 2, 3, 4 h) of cellular uptake of (A) mPDA and (B) MM@mPDA in RAW264.7 cells.


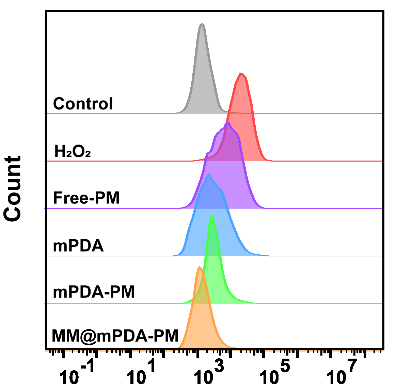


**Figure S4.** Flow cytometry analysis of DCFH-DA staining after different treatment.


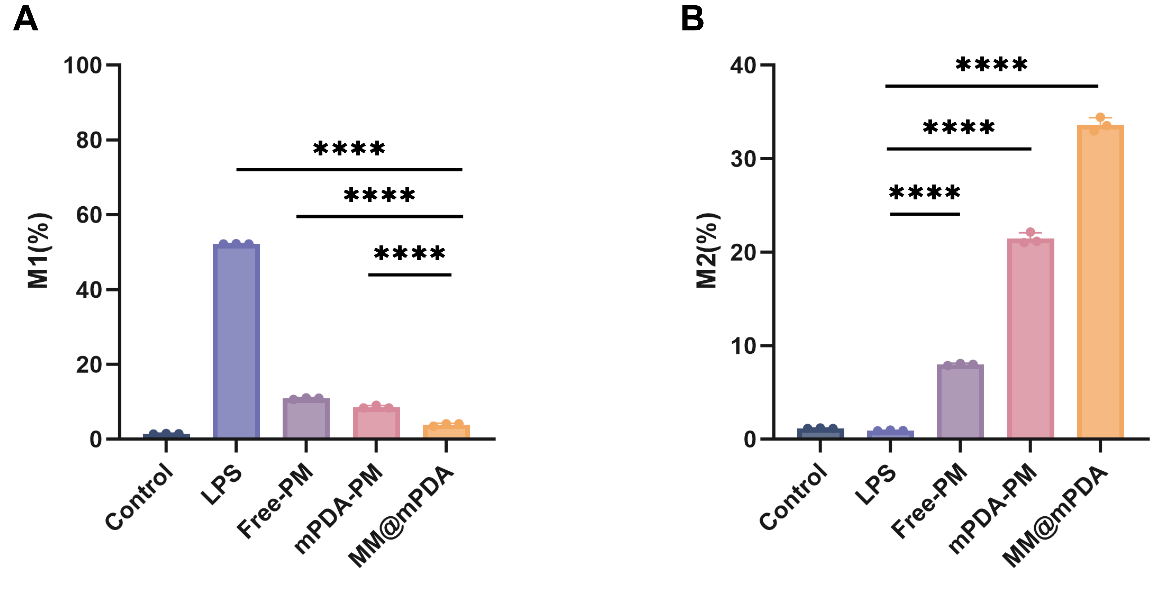


**Figure S5.** (A) Quantitative content of M1 rations of the raw cells. (B) Quantitative content of M2 rations of the raw cells. (n = 6 ) Data are expressed as the mean ± SD. Data were assessed using one-way ANOVA and two-way ANOVA; ****P < 0.0001.


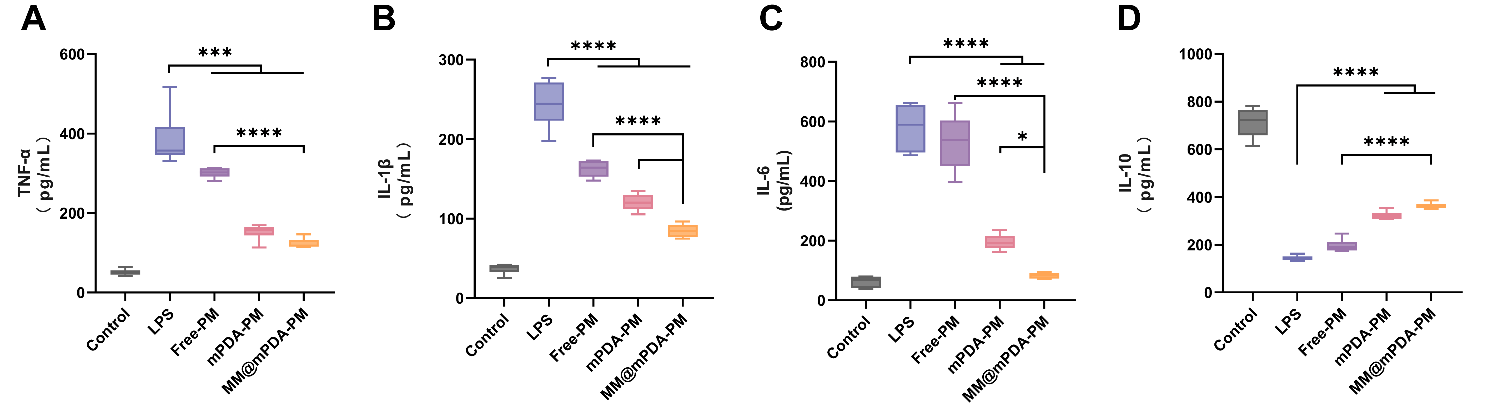


**Figure S6.** (A) TNF-α, (B) IL-1β, (C) IL-6 and (D)IL-10 levels in blood serum of different groups (n = 6). Data are expressed as the mean ± SD. Data were assessed using one-way ANOVA and two-way ANOVA; *P < 0.05, ***P < 0.001，****P < 0.0001.
